# Supplementary material for: In vitro production of cat-restricted Toxoplasma pre-sexual stages
Source: Nature. 2023 Dec 13;625(7994):366–76. doi: 10.1038/s41586-023-06821-y (PMC10781626; doi:10.1038/s41586-023-06821-y)
Supplement: Supplementary file 1 — Legends for Supplementary Tables 1–5. [file 41586_2023_6821_MOESM1_ESM.pdf]

nature portfolio

https://doi.org/10.1038/s41586-023-06821-y

Supplementary information

In vitro production of cat-restricted *Toxoplasma* pre-sexual stages

In the format provided by the authors and unedited

Supplementary Information guide

In vitro production of cat-restricted *Toxoplasma* pre-sexual stages by epigenetic reprogramming

Ana Vera Antunes[1]*, Martina Shahinas[1]*, Christopher Swale[1]*, Dayana C. Farhat[1], Chandra Ramakrishnan[3], Christophe Bruley[4], Dominique Cannella[1], Marie G. Robert[1], Charlotte Corrao[1], Yohann Couté[4], Adrian B. Hehl[3], Alexandre Bougdour[1], Isabelle Coppens[5], and Mohamed-Ali Hakimi[1] @

Supplementary Tables

A total of 5 tables are submitted as separate SI Excel files and their legend are included here.

Supplementary Table legends

Supplementary Table 1 | Description of *T. gondii* Strains, Plasmids, Primers and DNA synthesis. List of *T. gondii* parasite lines as well as plasmids used in this work. Primers and DNA synthesis construct used in this work are also charted in the table.

Supplementary Table 2 | AP2XII-1- and APX-2-regulated transcriptomes. Gene expression profiles in HFF showing that *T. gondii* genes are differentially regulated by AP2XII-1 and AP2XI-2 using different KD and conditions of IAA induction. RAW and TPM values are given for the indicated samples.

Supplementary Table 3 | MS-based quantitative analysis of total proteome from T. gondii depleted or not for AP2XII-1 and AP2XI-2. The proteomes from the AP2XII-1 KD / AP2XI-2 KD strain infecting HFF cells and treated (T) or not (UT) with IAA for 24h, 32h and 48h were analyzed by MS-based label-free quantitative proteomics (three biological replicates per condition). The quantification of proteins (log2 of filtered, normalized and imputed abundances of the different proteins in the different samples are given in columns O to Z) was based on razor and specific peptides (values indicated in column G). Statistical significance was tested using limma for two-by-two sample comparisons ; differentially abundant proteins were defined by a log2(fold change) ≥ 1 or ≤ -1 and a p-value ≤ 0.01, allowing to reach a false-discovery rate < 5 % according to the Benjamini-Hochberg estimator. A global comparison of the abundances of each protein in the four analyzed conditions was performed using ANOVA (column N).

Supplementary Table 4 | MS-based characterization of AP2XII-1 and AP2XI-2 interactomes. Flag immunoprecipitation eluates from HFF cells infected by *T. gondii* stably expressing HAFlag-tagged AP2XII-1 protein, HAFlag-tagged AP2XI-2 protein or none (Mock) were analyzed by MS-based label-free quantitative proteomics (three biological replicates per condition). Only proteins quantified with a minimum of five peptides, identified by MS/MS and quantified in all the replicates of one condition were considered. The quantification of proteins (log2 of filtered, normalized and imputed abundances of each protein in the different samples are given in columns O to W) was based on razor and specific peptides (the number of used peptides for each protein is indicated in column F). Statistical significance was tested using limma for two-by-two condition comparisons ; when comparing individual AP2 eluates with Mock eluates, differentially-abundant proteins were defined by a fold change ≥ 5 and a p-value ≤ 0.01, allowing to reach a false-discovery rate < 1 % according to the Benjamini-Hochberg estimator ; when comparing AP2 eluates with each other, differentially-abundant proteins were defined by a fold change ≥ 3 and a p-value ≤ 0.01, allowing to reach a false-discovery rate < 1 % according to the Benjamini-Hochberg estimator. The relative abundance relative to the bait protein of each protein found enriched with it compared to Mock was calculated based on iBAQ metrics (columns M and N); only enriched proteins showing an iBAQ ratio superior to 0.1 with respect to the bait protein were considered.

Supplementary Table 5 | MS-based characterization of AP2XI-2 interactome using protein expressed in insect cells. Total protein extracts from insect cells expressing AP2XI-2-Flag and AP2XII-1-(Strep)2 were submitted to FLAG immunoprecipitation. The eluates were then submitted to size-exclusion chromatography and the proteins in each fraction were separated by SDS-PAGE before staining with Coomassie blue. The protein content in the major protein band of the two main fractions was analyzed by MS-based proteomics. The results obtained for each band are presented in the corresponding data sheets.
