# Supplementary material for: In vitro production of cat-restricted Toxoplasma pre-sexual stages
Source: Nature. 2023 Dec 13;625(7994):366–76. doi: 10.1038/s41586-023-06821-y (PMC10781626; doi:10.1038/s41586-023-06821-y)
Supplement: Supplementary file 2 — Reporting Summary [file 41586_2023_6821_MOESM2_ESM.pdf]

nature portfolio

Corresponding author(s): HAKIMI MOHAMED-ALI

Last updated by author(s): 2023/09/17

Reporting Summary

Nature Portfolio wishes to improve the reproducibility of the work that we publish. This form provides structure for consistency and transparency in reporting. For further information on Nature Portfolio policies, see our Editorial Policies and the Editorial Policy Checklist.

Statistics

For all statistical analyses, confirm that the following items are present in the figure legend, table legend, main text, or Methods section.

n/a	Confirmed	
☐	☒	The exact sample size (*n*) for each experimental group/condition, given as a discrete number and unit of measurement
☒	☐	A statement on whether measurements were taken from distinct samples or whether the same sample was measured repeatedly
☐	☒	The statistical test(s) used AND whether they are one- or two-sided<br>*Only common tests should be described solely by name; describe more complex techniques in the Methods section.*
☒	☐	A description of all covariates tested
☒	☐	A description of any assumptions or corrections, such as tests of normality and adjustment for multiple comparisons
☐	☒	A full description of the statistical parameters including central tendency (e.g. means) or other basic estimates (e.g. regression coefficient) AND variation (e.g. standard deviation) or associated estimates of uncertainty (e.g. confidence intervals)
☐	☒	For null hypothesis testing, the test statistic (e.g. *F*, *t*, *r*) with confidence intervals, effect sizes, degrees of freedom and *P* value noted<br>*Give P values as exact values whenever suitable.*
☒	☐	For Bayesian analysis, information on the choice of priors and Markov chain Monte Carlo settings
☒	☐	For hierarchical and complex designs, identification of the appropriate level for tests and full reporting of outcomes
☐	☒	Estimates of effect sizes (e.g. Cohen's *d*, Pearson's *r*), indicating how they were calculated

Our web collection on statistics for biologists contains articles on many of the points above.

Software and code

Policy information about availability of computer code

Data collection	Librairies were sequenced on an Illumina NovaSeq platform and collected in FastQ format. MS and MS/MS data were acquired using Xcalibur software version 4.0 (Thermo Scientific). Peptides and proteins were identified using Mascot (version 2.6).
Data analysis	Integrated Genome Browser - by Nowlan H. Freese, David C. Norris, and Ann E. Loraine, Bioinformatics 2016 Jul 15;32(14):2089-95.<br>GraphPad - Prism 7<br>R (version 3.5.3), R Development Core Team, 2012<br>blastp (version 2.2.31+), Altschul et al., 1990<br>BOWTIE (V2.1.0)<br>Samtools v1.4<br>Bamtools v2.5.1<br>Guppy v5 or higher<br>minimap2 v2.17<br>Deeptools v3.5.1 was used for global peak intensity over gene features.<br>Basecaller software (OLB V1.8)<br>SeqMan NGen (version 14, DNASTAR. Madison, WI, USA)<br>Lasergene Genomics Suite version 14 (DNASTAR, Madison, WI, USA)<br>ArrayStar module (version 14, DNASTAR. Madison, WI, USA)<br>ClustalW software<br>RAW files were processed using MaxQuant version 1.6.2.10.<br>Proline software (http://proline.profiproteomics.fr) was used to filter the results.<br>Statistical analyses were performed using ProStaR (Wieczorek, S. et al. DAPAR & ProStaR: software to perform statistical analyses in

quantitative discovery proteomics. Bioinformatics 33, 135–136 (2017).
nf-core ATAC-SEQ v2.0 is a nextflow pipeline for ATAQ-SEQ data processing
Integrated Genome Browser (IGB) v9.1.8 or higher

For manuscripts utilizing custom algorithms or software that are central to the research but not yet described in published literature, software must be made available to editors and reviewers. We strongly encourage code deposition in a community repository (e.g. GitHub). See the Nature Portfolio guidelines for submitting code & software for further information.

Data

Policy information about availability of data

All manuscripts must include a data availability statement. This statement should provide the following information, where applicable:

- Accession codes, unique identifiers, or web links for publicly available datasets
- A description of any restrictions on data availability
- For clinical datasets or third party data, please ensure that the statement adheres to our policy

The ChIP-seq data have been deposited to the GEO Dataset :
https://www.ncbi.nlm.nih.gov/geo/query/acc.cgi?acc=GSE222819

The ATAC-seq data have been deposited to the GEO Dataset :
https://www.ncbi.nlm.nih.gov/geo/query/acc.cgi?acc=GSE222832

The Nanopore and Illumina RNAseq data have been deposited to the GEO Datasets under the BioProject: PRJNA921935
https://www.ncbi.nlm.nih.gov/bioproject/PRJNA921935

MS-base proteomic data have been deposited to the ProteomeXchange Consortium via the PRIDE partner repository with the dataset identifiers PXD039400 and PXD042658 for respectively proteome-wide and interactomic analyses.
https://www.ebi.ac.uk/pride/archive/projects/PXD039400
https://www.ebi.ac.uk/pride/archive/projects/PXD042658

Human research participants

Policy information about studies involving human research participants and Sex and Gender in Research.

Reporting on sex and gender	N/A
Population characteristics	N/A
Recruitment	N/A
Ethics oversight	N/A

Note that full information on the approval of the study protocol must also be provided in the manuscript.

Field-specific reporting

Please select the one below that is the best fit for your research. If you are not sure, read the appropriate sections before making your selection.

☒ Life sciences ☐ Behavioural & social sciences ☐ Ecological, evolutionary & environmental sciences

For a reference copy of the document with all sections, see nature.com/documents/nr-reporting-summary-flat.pdf

Life sciences study design

All studies must disclose on these points even when the disclosure is negative.

Sample size	Sample sizes were determined because differences among groups were consistent and significant.<br>All experiments were performed in biological replicates to allow for statistical analyses.
Data exclusions	No data were excluded from the analysis
Replication	All findings were reproduced successfully, and replications are described in methods and figure legends in more detail.
Randomization	No method of randomization was used and all experiments were performed in independent biological replicates as stated for each experiment in the main manuscript. All corresponding treatment and mock samples were processed at the same time to minimize technical variation. Random images of cells were evaluated for IFA and TEM. Randomization for other experiments were not subjective.

Blinding	Investigators were not blinded during the experiments. Experiments were performed in biological replicates and provided consistent statistically relevant results. Other experiments were not performed with blinding since no subjective evaluations were involved.

Reporting for specific materials, systems and methods

We require information from authors about some types of materials, experimental systems and methods used in many studies. Here, indicate whether each material, system or method listed is relevant to your study. If you are not sure if a list item applies to your research, read the appropriate section before selecting a response.

Materials & experimental systems		
n/a	Involved in the study	
☐	☒	Antibodies
☒	☐	Eukaryotic cell lines
☒	☐	Palaeontology and archaeology
☐	☒	Animals and other organisms
☒	☐	Clinical data
☒	☐	Dual use research of concern

Methods		
n/a	Involved in the study	
☐	☒	ChIP-seq
☒	☐	Flow cytometry
☒	☐	MRI-based neuroimaging

Antibodies

Antibodies used	The following primary antibodies were used in the immunofluorescence, immunoblotting, and/or ChIP assays: rabbit anti-TgHDAC3 (RRID: AB_2713903), rabbit anti-TgGAP45 (gift from Pr. Dominique Soldati), mouse anti-HA tag (Roche, RRID: AB_2314622), rabbit anti-HA Tag (Cell Signaling Technology, RRID: AB_1549585), rabbit anti-mCherry (Cell Signaling Technology, RRID: AB_2799246), rabbit anti-FLAG (Cell Signaling Technology, RRID: AB_2798687), mouse anti-MYC clone 9B11 (RRID: AB_2148465), H3K9me3 (Diagenode, RRID: AB_2616044), rabbit Anti-acetyl-Histone H4, pan (Lys 5,8,12) (Millipore, RRID:AB_310270), rat anti-IMC7 (gift from Pr. Gubbels MJ), mouse anti-IMC1 (gift from Pr. Ward GE), mouse anti-AtRx antibody clone 11G826, mouse anti-GRA11b14. We have also raised homemade antibodies against linear peptides in rabbits corresponding to the following proteins: MORC_Peptide2 (C +SGAPIWTGERGSGA); AP2XI-2 (C+HAFKTRRTEAAT) TGME49_273980/GRA80 (C+RPPWAPGAGPEN); TGME49_243940/GRA81 (C +QKELAEVAQRALEN); TGME49_277230/GRA82 (C+SDVNTEGDATVANPE); TGME49_209985/ROP26 (CQETVQGNGETQL); SRS48 family (CKALIEVKGVPK); SRS59B/K (C+IHVPGTDSTSSGPGS); TGME49_314250/BRP1 (C+QVKEGTKNNKGLSDK); TGME49_307640/CK2 kinase (C+IRAQYHAYKGKYSHA); and TGME49_306455 (C+DGRTPVDRVFEE). They were manufactured by Eurogentec and used for immunofluorescence, immunoblotting and/or chromatin immunoprecipitation. Secondary immunofluorescent antibodies were coupled with Alexa Fluor 488 or Alexa Fluor 594 (Thermo Fisher Scientific). Secondary antibodies used in Western blotting were conjugated to alkaline phosphatase (Promega) or horseradish peroxidase.
Validation	All the antibodies used in this study were validated for ChIP-seq, IFA or Western blot by several groups according to their RRID numbers and sometimes by the company that own them. GAP45, IMC7, IMC1, AtRx(11G8) and GRA11b are well known antibody in Toxoplasma gondii and were validated by several publications.

Animals and other research organisms

Policy information about studies involving animals; ARRIVE guidelines recommended for reporting animal research, and Sex and Gender in Research

Laboratory animals	Six-week-old NMRI, CD1 or Balb/C mice were obtained from Janvier Laboratories (Le Genest-Saint-Isle, France)
Wild animals	No wild animals were used in this study
Reporting on sex	Female mice were used for all studies.
Field-collected samples	No field-collected samples were used in this study
Ethics oversight	Mouse care and experimental procedures were performed under pathogen-free conditions in accordance with established institutional guidance and approved protocols from the Institutional Animal Care and Use Committee of the University Grenoble Alpes (APAFIS#4536-2016031 017075121 v5).

Note that full information on the approval of the study protocol must also be provided in the manuscript.

ChIP-seq

Data deposition

☒ Confirm that both raw and final processed data have been deposited in a public database such as GEO.

☒ Confirm that you have deposited or provided access to graph files (e.g. BED files) for the called peaks.

Data access links
May remain private before publication.

The ChIP-seq data have been deposited to the GEO Dataset :
Series GSE222819
To review GEO accession GSE222819:
Go to https://www.ncbi.nlm.nih.gov/geo/query/acc.cgi?acc=GSE222819
Enter token wjefcuwirhybtqh into the box

Files in database submission

GSM6932717 AP2-XII-1-KD-HA_HA-antibody_UT
GSM6932718 AP2-XII-1-KD-HA_HA-antibody_IAA24h
GSM6932719 AP2-XII-1-KD-HA_HDAC3-antibody_UT
GSM6932720 AP2-XII-1-KD-HA_HDAC3-antibody_IAA24h
GSM6932721 AP2-XII-1-KD-HA_MORC-antibody_UT
GSM6932722 AP2-XII-1-KD-HA_MORC-antibody_IAA24h
GSM6932723 AP2-XI-2-KD-HA_HA-antibody_UT
GSM6932724 AP2-XI-2-KD-HA_HA-antibody_IAA24h
GSM6932725 AP2-XI-2-KD-HA_HDAC3-antibody_UT
GSM6932726 AP2-XI-2-KD-HA_HDAC3-antibody_IAA24h
GSM6932727 AP2-XI-2-KD-HA_MORC-antibody_UT
GSM6932728 AP2-XI-2-KD-HA_MORC-antibody_IAA24h
GSM6932729 AP2-XII-1-KD-HA_AP2-XI-2-KD-Myc_HA-antibody_UT
GSM6932730 AP2-XII-1-KD-HA_AP2-XI-2-KD-Myc_HA-antibody_IAA24h
GSM6932731 AP2-XII-1-KD-HA_AP2-XI-2-KD-Myc_Myc-antibody_UT
GSM6932732 AP2-XII-1-KD-HA_AP2-XI-2-KD-Myc_Myc-antibody_IAA24h
GSM6932733 AP2-XII-1-KD-HA_AP2-XI-2-KD-Myc_HDAC3-antibody_UT
GSM6932734 AP2-XII-1-KD-HA_AP2-XI-2-KD-Myc_HDAC3-antibody_IAA24h
GSM6932735 AP2-XII-1-KD-HA_AP2-XI-2-KD-Myc_MORC-antibody_UT
GSM6932736 AP2-XII-1-KD-HA_AP2-XI-2-KD-Myc_MORC-antibody_IAA24h

Genome browser session
(e.g. UCSC)

Not applicable

Methodology

Replicates

MORC, HDAC3 or HA : 3 different Chips in different parental strains

Sequencing depth

Sequencing layout: 1x150bp
Sequencing Depth for each sample ID
ID: total number of reads (Pass solexa CHASTITY quality filter)/uniquely mapped (ToxoDB-13.0_TgondiiME49)
GSM6932717 AP2-XII-1-KD-HA_HA-antibody_UT (47,375,300/16,108,744 reads)
GSM6932718 AP2-XII-1-KD-HA_HA-antibody_IAA24h (33,607,612/11,284,027 reads)
GSM6932719 AP2-XII-1-KD-HA_HDAC3-antibody_UT (41,375,218/17,389,398 reads)
GSM6932720 AP2-XII-1-KD-HA_HDAC3-antibody_IAA24h (40,312,162/14,737,507 reads)
GSM6932721 AP2-XII-1-KD-HA_MORC-antibody_UT (37,349,606/10,598,146 reads)
GSM6932722 AP2-XII-1-KD-HA_MORC-antibody_IAA24h (34,730,526/9,808,916 reads)
GSM6932723 AP2-XI-2-KD-HA_HA-antibody_UT (38,347,182/8,439,251 reads)
GSM6932724 AP2-XI-2-KD-HA_HA-antibody_IAA24h (30,766,515/6,807,339 reads)
GSM6932725 AP2-XI-2-KD-HA_HDAC3-antibody_UT (37,024,267/10,518,299 reads)
GSM6932726 AP2-XI-2-KD-HA_HDAC3-antibody_IAA24h (35,824,007/11,383,677 reads)
GSM6932727 AP2-XI-2-KD-HA_MORC-antibody_UT (32,639,068/7,847,338 reads)
GSM6932728 AP2-XI-2-KD-HA_MORC-antibody_IAA24h (34,919,949/9,531,074 reads)
GSM6932729 AP2-XII-1-KD-HA_AP2-XI-2-KD-Myc_HA-antibody_UT (26,209,227/6,862,246 reads)
GSM6932730 AP2-XII-1-KD-HA_AP2-XI-2-KD-Myc_HA-antibody_IAA24h (26,015,064/5,812,766 reads)
GSM6932731 AP2-XII-1-KD-HA_AP2-XI-2-KD-Myc_Myc-antibody_UT (17,571,565/3,432,148 reads)
GSM6932732 AP2-XII-1-KD-HA_AP2-XI-2-KD-Myc_Myc-antibody_IAA24h (28,627,661/6,737,545 reads)
GSM6932733 AP2-XII-1-KD-HA_AP2-XI-2-KD-Myc_HDAC3-antibody_UT (19,248,893/4,861,975 reads)
GSM6932734 AP2-XII-1-KD-HA_AP2-XI-2-KD-Myc_HDAC3-antibody_IAA24h (23,609,829/3,008,898 reads)
GSM6932735 AP2-XII-1-KD-HA_AP2-XI-2-KD-Myc_MORC-antibody_UT (20,981,524/4,634,579 reads)
GSM6932736 AP2-XII-1-KD-HA_AP2-XI-2-KD-Myc_MORC-antibody_IAA24h (19,924,121/3,299,573 reads)

Antibodies

rabbit anti-TgHDAC3 (Bougdour et al., 2009; RRID: AB_2713903)
rabbit anti-HA Tag (Cell Signaling Technology, RRID: AB_1549585)
mouse anti-MYC clone 9B11 (RRID: AB_2148465)
rabbit MORC_Peptide2 (C+SGAPIWTGERGSGA)

Peak calling parameters

After the sequencing platform generated the sequencing images, the stages of image analysis and base calling were performed using Off-Line Basecaller software (OLB V1.8). After passing Solexa CHASTITY quality filter, the clean reads were aligned to T. gondii reference genome (TGME49) using BOWTIE V2 then converted and sorted using Bamtools V2.5. Aligned reads were used for peak calling of the ChIP enriched peaks using MACS V2.2 with a cutoff p-value of 10-4. Data visualization: For IGB visualization and gene centered analysis using Deeptools, MACS2 generated bedgraph files were processed with the following command: “sort -k1,1 -k2,2n 5_treat_pileup.bdg > 5_treat_pileup-sorted.bdg” then converted using the BedGraphToBigWig program (ENCODE project). The Deeptools analysis were generated using “computeMatrix reference point” with the following parameters (--minThreshold 2, --binSize 10 and –averageTypeBins sum). Plotprofile or heatmap was then used with a k-mean clustering when applicable. Inter sample comparison were obtained using the nf-core chip-seq V2.0.0 workflow with standard parameters. From this pipeline, HOMER

(annotatePeaks) was used to analyze peak distribution relative to gene features. All these raw and processed files can be found at Series GSE222819.

Data quality

Raw_data: Contains all reads which pass Solexa CHASTITY quality filter in FASTQ format (*_sequence.fastq files).

Software

BOWTIE2 software (V2.1.0)
MACS v2.2 (Model-based Analysis of ChIP-seq) software was used to detect the peak from ChIP-seq data.
Deeptools v3.5.1 was used for global peak intensity over gene features.
nf-core Chip-Seq v2.0.0 is a nextflow pipeline for Chip-Seq analysis.
Samtools v1.4
Bamtools v2.5.1
ucsc-bedgraphtobigwig v377
Integrated Genome Browser (IGB) v9.1.8 or higher
