# Supplementary material for: In vitro production of cat-restricted Toxoplasma pre-sexual stages
Source: Nature. 2023 Dec 13;625(7994):366–76. doi: 10.1038/s41586-023-06821-y (PMC10781626; doi:10.1038/s41586-023-06821-y)
Supplement: Supplementary file 11 — Source Data Fig. 5 [file 41586_2023_6821_MOESM11_ESM.pdf]

Figure 5b

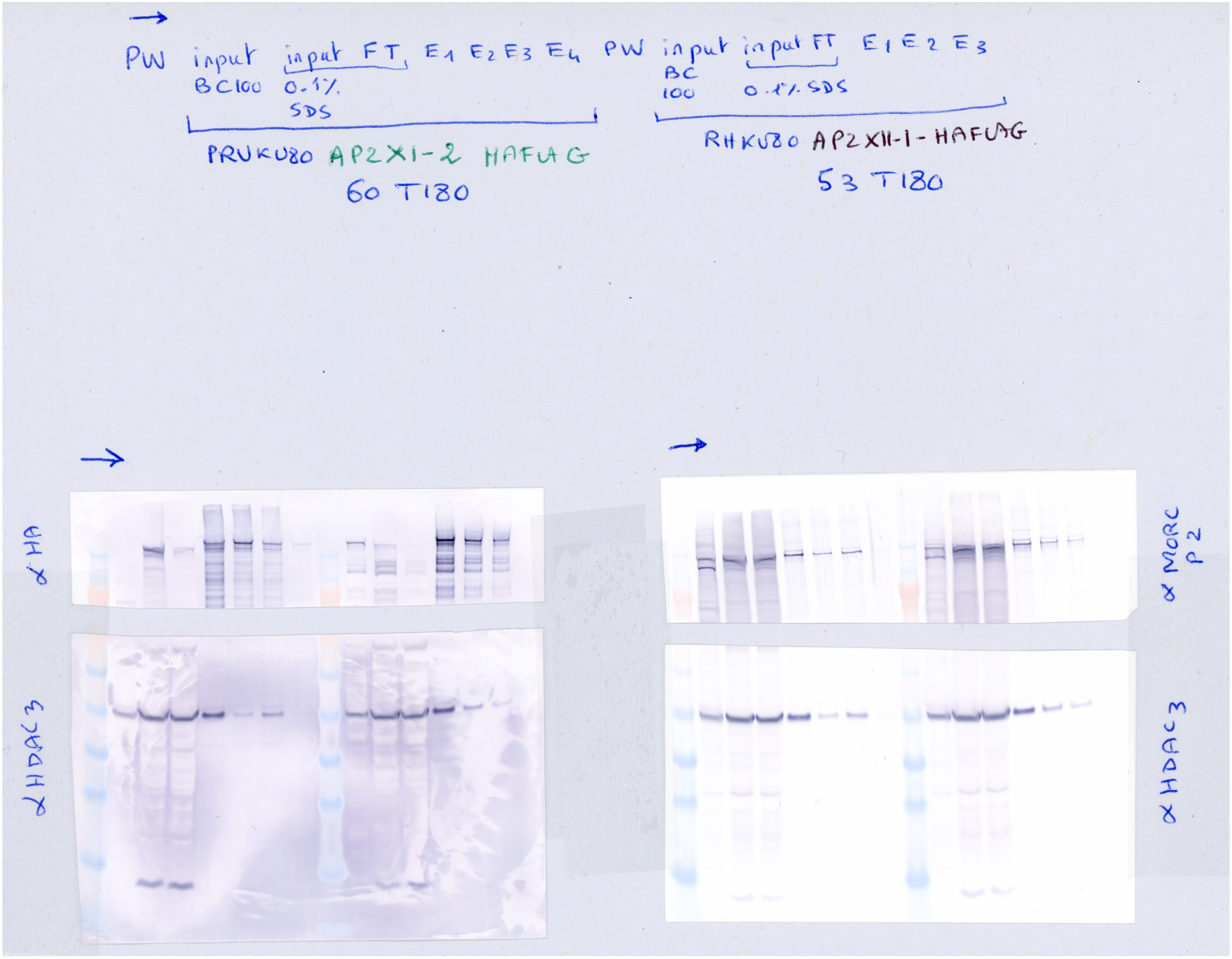

PW input input FT E1 E2 E3 E4
BC100 0.1% SDS
PRUKU80 AP2XI-2 HAFLAG
60 T180
PW input input FT E1 E2 E3
BC 100 0.1% SDS
RHKU80 AP2XII-1-HAFLAG
53 T180
α HA
α HDAC3
α MORC P2
α HDAC3

Figure 5d

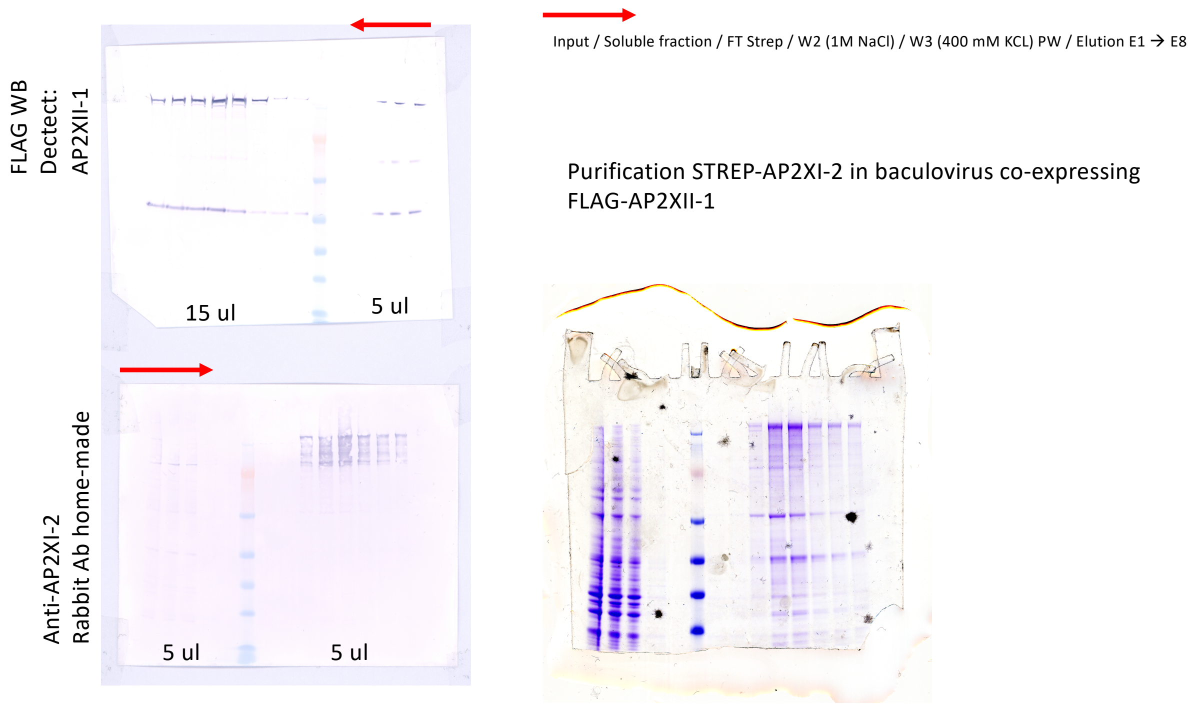

Figure 5e

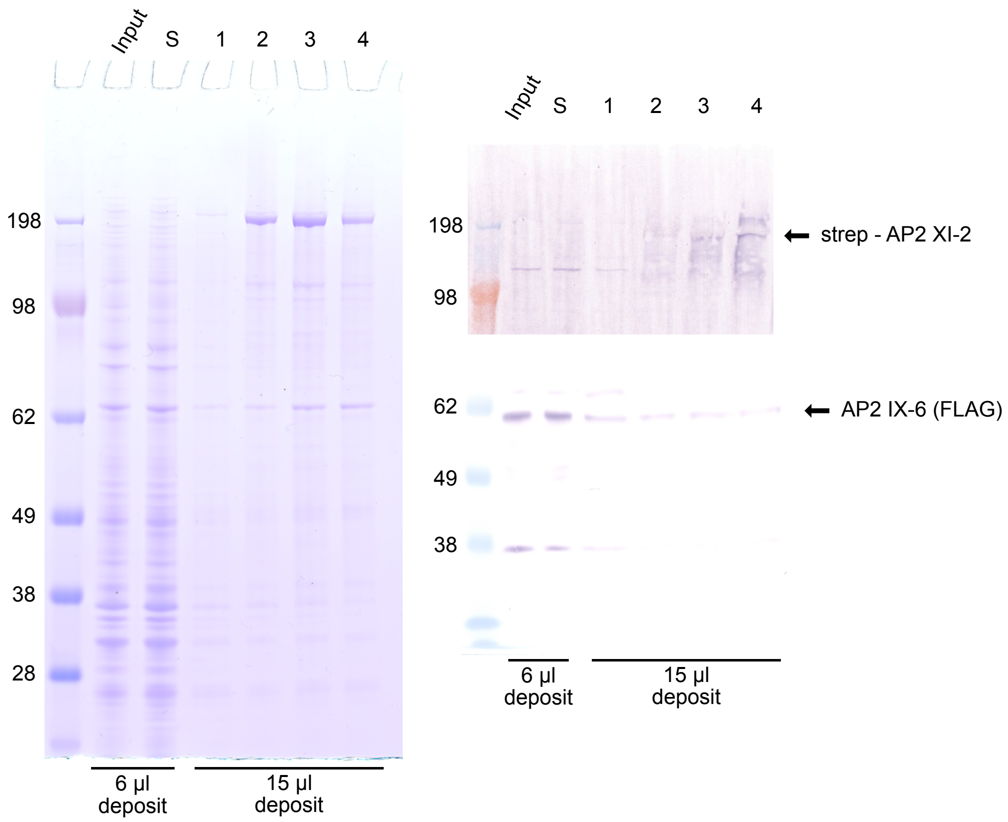

4-12% NuPAGE run in MES buffer and colored with Coomassie blue dye.

Figure 5f

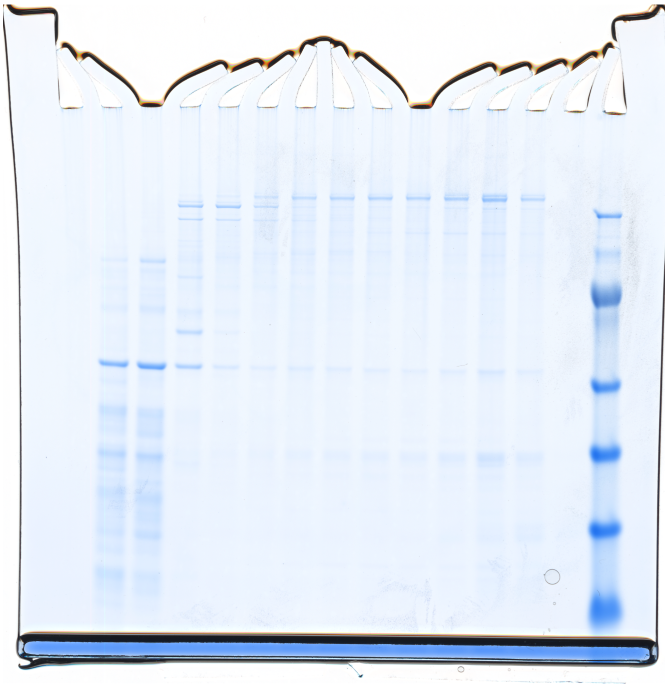

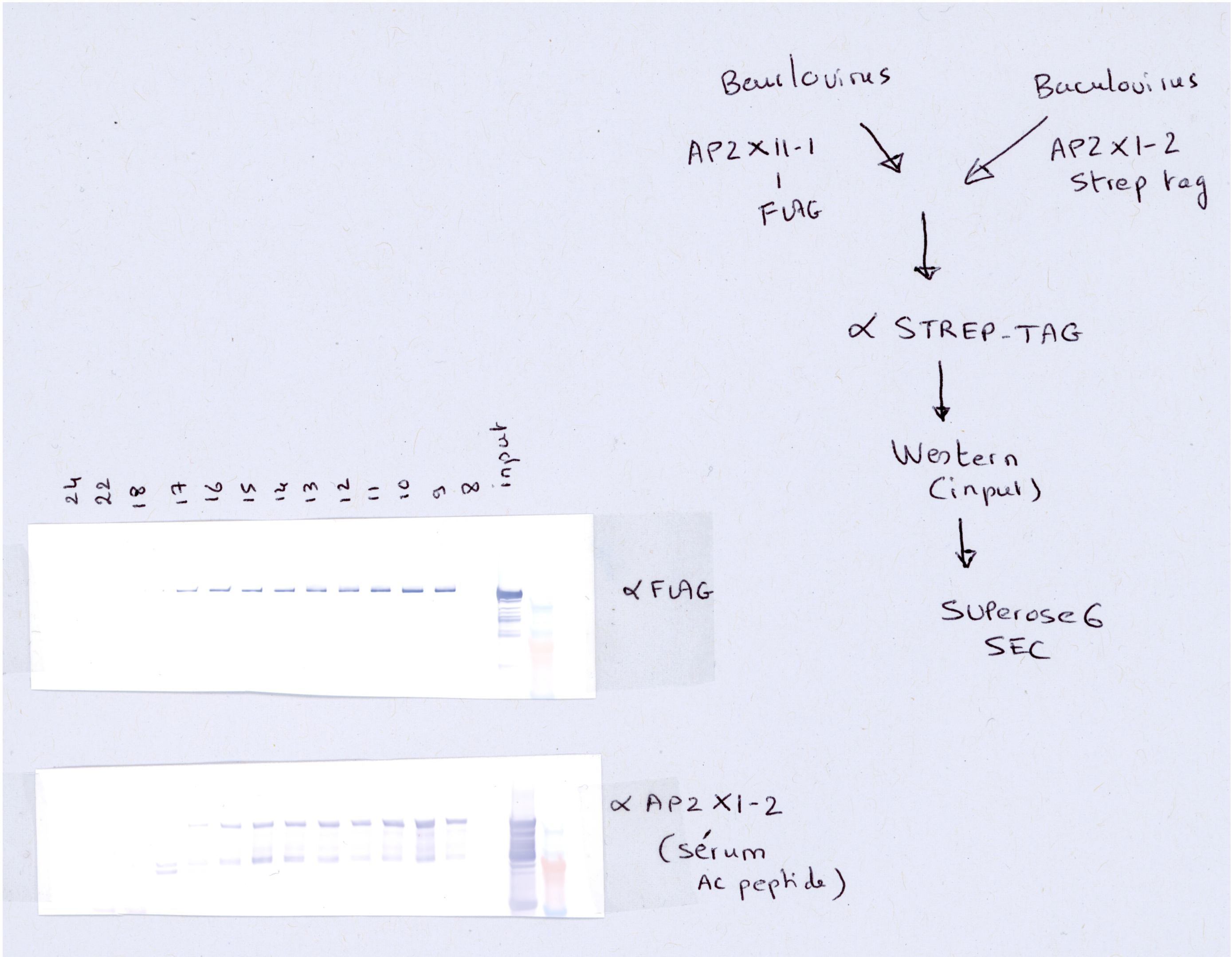
Baculovirus
AP2XII-1
FLAG
Baculovirus
AP2XI-2
Strep tag
α STREP-TAG
Western
(input)
Superose6
SEC
24 22 18 17 16 15 14 13 12 11 10 9 8 input
αFLAG
α AP2XI-2
(sérum
Ac peptide)
